# Tumor-infiltrating immune cells and prognosis in gastric cancer: a systematic review and meta-analysis

**DOI:** 10.18632/oncotarget.17602

**Published:** 2017-05-03

**Authors:** Wen Jiang, Ke Liu, Qing Guo, Ji Cheng, Liming Shen, Yinghao Cao, Jing Wu, Jianguo Shi, Heng Cao, Bo Liu, Kaixiong Tao, Guobin Wang, Kailin Cai

**Affiliations:** ^1^ Department of Gastrointestinal Surgery, Union Hospital, Tongji Medical College, Huazhong University of Science and Technology, Wuhan, China; ^2^ Department of Pediatrics, Union Hospital, Tongji Medical College, Huazhong University of Science and Technology, Wuhan, China; ^3^ MOE Key Lab of Environment and Health, School of Public Health, Tongji Medical College, Huazhong University of Science and Technology, Wuhan, China; ^4^ Department of Maternal and Child Health, School of Public Health, Tongji Medical College, Huazhong University of Science and Technology, Wuhan, China; ^5^ Department of Gastrointestinal Surgery, The Central Hospital of Wuhan, Tongji Medical College, Huazhong University of Science and Technology, Wuhan, China; ^6^ Department of Gastrointestinal Surgery, Xinyang Central Hospital, Xinyang, China

**Keywords:** gastric cancer, prognosis, tumor-infiltrating immune cells, overall survival, meta-analysis

## Abstract

Tumor-infiltrating immune cells are a pivotal component of the tumor microenvironment (TME), but their indicative role remains poorly defined. A meta-analysis was performed to reveal the prognostic efficiency of tumor-infiltrating immune cells in gastric cancer (GC). By searching PubMed and Embase, we identified a total of 35 eligible articles that involved 4888 patients. Random or fixed effect models were employed to extract pooled hazard ratios (HRs) with 95% confidence intervals (CIs). Our results indicated that high CD3+ lymphocyte infiltration in all the locations (AG), the tumor nest (TN), and the tumor stroma (TS) predicted better overall survival (OS) (HR=0.71, 95% CI=0.57-0.90; HR=0.58, 95% CI=0.42-0.80; and HR=0.50, 95% CI=0.37-0.68, respectively). CD8+ T cell infiltration in AG and FoxP3+ regulatory T cells (Tregs) in the tumor invasive margin (TM) were also associated with improved OS (HR=0.90, 95% CI=0.83-0.97; HR=0.65, 95% CI=0.48-0.87, respectively). However, contrasting results were found in the macrophage subset, with M2 in AG (HR=1.45, 95% CI=1.13-1.86) and the TN (HR=1.67, 95% CI=1.12-2.48) associated with worse OS. In summary, the combination of the densities and locations of tumor-infiltrating immune cells can be useful for predicting survival for GC patients, but additional research is needed to reinforce the reliability of this study’s conclusions.

## INTRODUCTION

Gastric cancer (GC) is one of the most common malignancies. Its incidence and mortality rates ranked fifth and second in 2013, respectively, placing a heavy burden on the public health system worldwide, especially in East Asian countries [1, 2]. Diagnosis and treatment strategies are based on the TNM staging system, which has been revised and perfected over the past 80 years. However, the prognosis of GC can be affected by several factors, such as tumor volume, patient age, and nutrition status. Thus, GC patients with the same TNM stage can have different clinical outcomes, causing unreliability in the TNM staging system for prognosis assessments. A new method to improve the accuracy of the TNM staging system is urgently needed.

Immune cells are a major component of the tumor microenvironment and come in multiple types with different functions. CD3 is a marker of T lymphocytes, including CD4+ T helper lymphocytes, CD8+ cytotoxic T lymphocytes, and FoxP3+ regulatory cells (Tregs). CD8+ T cells are cytotoxic and kill target tumor cells or promote tumor destruction via secretion of effector cytokines such as interferon-c or tumor necrosis factor [3, 4]. CD4+ helper T lymphocytes are required for the induction and maintenance of CD8+ T cells [5]. FoxP3+ Tregs suppress antitumor responses and maintain immunological tolerance to host tissues [6]. Similarly, tumor-associated macrophages (TAMs) can be divided into M1 (classically activated) and M2 (alternatively activated) cells. M2 cells promote tumor growth and progression and help subvert adaptive immunity [7]. However, recent reports have indicated that the presence of CD4+ helper T lymphocytes, FoxP3+ Tregs and M2 cells can lead to favorable outcomes in certain tumor patients [8-11]. Therefore, it is necessary to summarize the current progress regarding what is known of the relationship between tumor-infiltrating immune cells and the prognosis of cancer patients.

To date, the densities and locations of tumor-infiltrating immune cells have proven to be associated with clinical outcomes in lung cancer [12], colorectal cancer [13], breast cancer [14] and ovarian cancer [15], among others. Moreover, Galon et al [16] proposed that the type, density, and location of immune cells in colorectal cancer have prognostic values that are superior to and independent of those of the TNM classification. Nevertheless, the predictive role of tumor-infiltrating immune cells in patients with GC cancer remains controversial. Therefore, we performed a systematic review and meta-analysis to investigate the correlation between tumor-infiltrating immune cells and GC survival stratified according to immune cell subset and infiltration location (tumor nest, tumor stroma or tumor invasive margin).

## RESULTS

### Eligible studies

**Figure 1:**
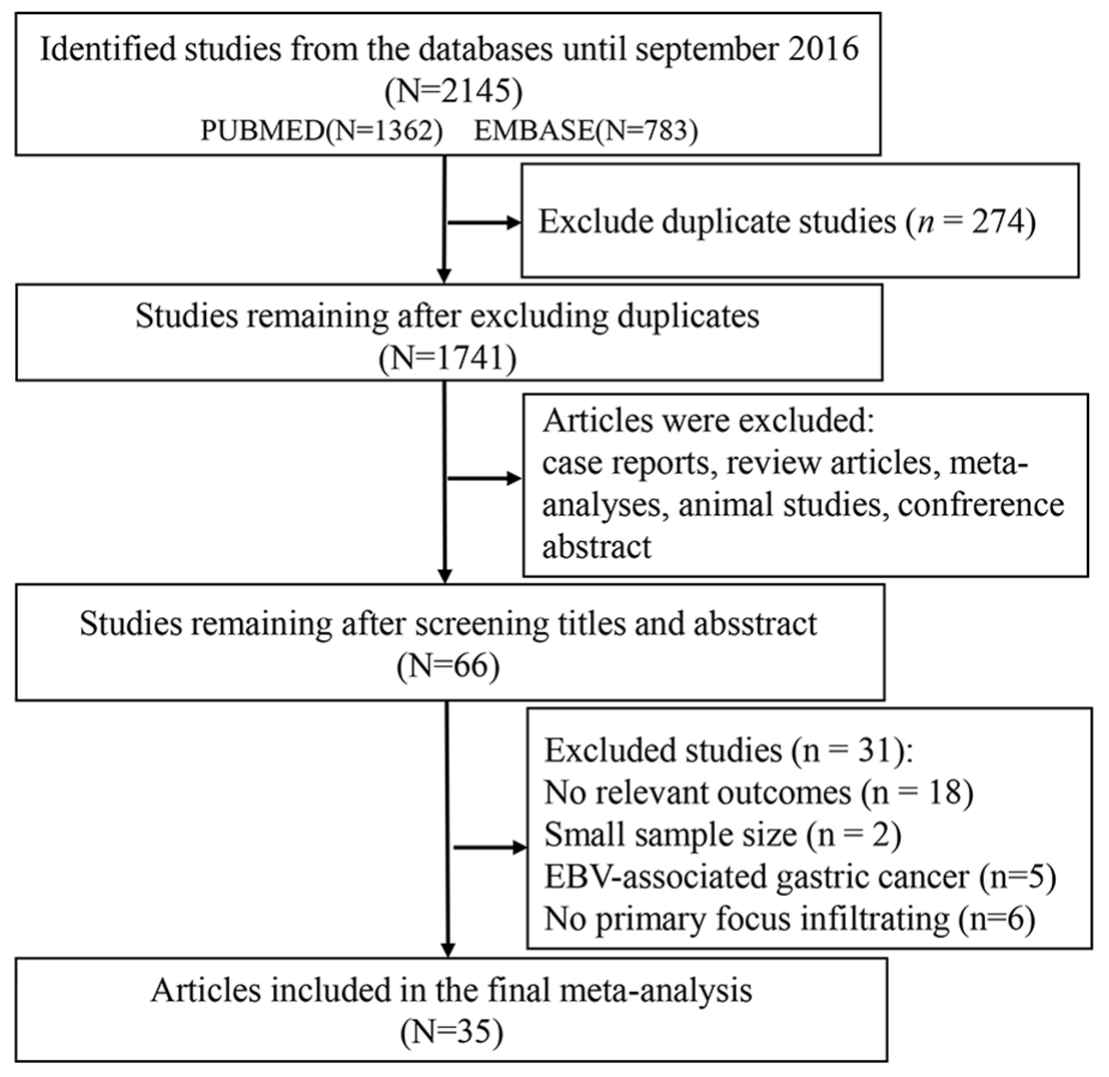
Flow chart for screening eligible publications

After screening, 35 articles were included in the meta-analysis (Figure 1). The basic characteristics of each study are presented in Table 1 and Supplementary Table 1 [9, 10, 17-49]. Among the 35 articles, 28 articles reported tumor-infiltrating lymphocytes, including CD3+ T cells (n=8), CD4+ helper T cells (n=6), CD8+ cytotoxic T cells (n=12), CD20+ B cells (n=2), CD45RO+ memory cells (n=2), FoxP3+ regulatory T cells (n=16), t-bet+ cells (n=2), dendritic cells (n=3), granzyme B cells (n=2), and natural killer cells (n=2). Twelve studies contained macrophages, which have two polarizations, M1 (n=2) and M2 (n=6). And CD11c/iNoS were identified as the marker of M1 and CD163/CD206 were identified as the marker of M2. The cell counting locations can mainly be divided into three categories: the tumor nest (TN), the tumor stroma (TS) and the tumor invasive margin (TM). In addition, in certain included articles, immune cells were counted without distinguishing among cell counting locations (such immune cell counts were incorporated into the data for all the location (AG)).

**Table 1:** Basic characteristics of eligible studies.

Author, Year	Region	Assay	Study design	N (male/female)	Cutoff point	Subsets	Location	Outcomes	Score
Zhang, 2016	China	IHC	Cohort	178(125/53)	Mean	M	AG	OS	6
Yan, 2016	China	IHC	Cohort	178(125/53)	Mean	M2	AG	OS	6
Park, 2016	Korea	IHC	Cohort	113(87/36)	Mean	M2	TN/TS/TM	OS/DFS	5
Li, 2016	China	IHC	Cohort	212(148/64)	Median	CD57	TN	DFS/OS	6
Kim, 2016	Korea	TMA	Cohort	243(152/91)	Median	CD3/CD4/CD8	AG	DFS	5
Kawazoe, 2016	Japan	IHC	Cohort	383	Median	CD3/CD4/CD8/Foxp3	AG	OS	6
Hennequin, 2016	France	IHC	Cohort	82(57/25)	Median	CD8/CD20/Foxp3/Tbet	TN/TS/TM	RFS	5
Giampieri, 2016	Italy	IHC	Cohort	73	50–60 % stromal area	CD3	TS	OS	4
Zhang, 2015	China	IHC	Cohort	180(56/124)	Median	M/M1/M2	TN	OS	6
Suh, 2015	Korea	IHC	Cohort	117	15/HPF	Foxp3	AG	DFS/OS	6
Liu, 2015	China	IHC	Cohort	166(125/41)	median	CD3/CD4/CD8/ Foxp3/CD57/M	TN/TS/TM	OS	7
Lin, 2015	China	IHC	Cohort	170(97/73)	Grade C	M2	AG	OS	3
Li, 2015	China	IHC	Cohort	192(138/54)	5% staining	CD4/CD8	AG	OS	5
Kim, 2015	Korea	IHC	Cohort	143	CD8/Foxps3 median M/M2 score 1	CD8/Foxp3/M/M2	TN/TS/ TM/AG	DFS/PFS	6
Geng, 2015	China	IHC	Cohort	100(61/39)	25% stainiing	Foxp3	AG	OS	6
Okita, 2014	Japan	IHC	Cohort	214(157/57)	Median	DC	AG	OS	4
Ma, 2014	China	IHC	Cohort	135(90/45)	>25/HPF high <5/HPF low.	Foxp3	IN	OS	5
Kim,2014	Korea	IHC	Cohort	99(55/44)	CD8/60th percentile Foxp3/Median	CD8/Foxp3	TN	OS	6
Arigami, 2014	Japan	IHC	Cohort	120(74/46)	Median	CD3	AG	OS	6
Zhou, 2013	China	IHC	Cohort	133(89/44)	Mean	Foxp3	AG	OS	6
Wakatsuki, 2013	Japan	IHC	Cohort	74(54/20)	Mean	CD45RO	AG	OS	4
Pantano, 2013	Italy	IF	Cohort	52(23/29)	Median	M1/M2	AG	OS	6
Chen, 2013	China	IHC	Cohort	152(117/35)	19.05/HPF	Tbet	AG	DFS/OS	5
Kashimura,2012	Japan	IHC	Cohort	123(89/34)	Mean	Foxp3/DC	AG	DFS/OS	5
Ishigami,2012	Japan	IHC	Cohort	141(92/36)	10/HPF	Foxp3	TS	OS	3
Wang, 2011	China	IHC	Cohort	107(69/38)	Median	Foxp3/M	TN/TM	OS	7
Kim,2011	Korea	IHC	Cohort	180(126/54)	Median	CD3/CD4/CD8/Foxp3/ Granzyme B	TN	OS/RFS	6
Shen, 2010	China	IHC	Cohort	133(89/44)	Median	CD4/CD8 /Foxp3	TN/TM	OS	6
Haas,2009	Germany	IHC	Cohort	52(40/12)	Median	CD3/CD8/CD20/Foxp3/ Granzyme B/M	TN/TS	OS	6
Perrone,2008	Italy	IHC	Cohort	110(53/57)	Median	Foxp3	TN	OS/RFS	4
Mizukami, 2008	Japan	IHC	Cohort	80(56/24)	Median	Foxp3	AG	OS	5
Lee, 2008	Korea	IHC	Cohort	220(156/64)	Mean	CD3/CD8/ CD45RO	AG	OS	6
Ohno,2005	Japan	IHC	Cohort	84(57/27)	median	CD8/M	TN/TM	DFS	6
Ohno,2003	Japan	IHC	Cohort	84(57/27)	median	M	TN	DFS	6
Takahashi,2002	Japan	IHC	Cohort	65(44/21)	20 positive cells	DC	AG	OS	3

Abbreviations: AG=all the location, TN=tumor nest, TS=tumor stroma, TM=tumor invasive margin, OS=overall survival, DFS=disease-free survival, RFS=relapse-free survival, IHC=immunohistochemistry, TMA=tissue microarrays, IF=immunofluorescence.

This meta-analysis included studies involving a total of 4888 patients from six countries, including China (n=13), France (n=1), Germany (n=1), Italy (n=3), Japan (n=10), and Korea (n=7). Nine studies included less than 100 patients, five articles contained more than 200 patients, and the remaining publications enrolled between 100 and 200 patients. The score of eligible articles ranged from 3 to 7, with 28 articles ≥5 and 7 articles <5. Hazard ratios (HRs) for overall survival (OS) and DFS/RFS (disease-free survival/relapse-free survival) of 5 articles were estimated through survival curves. The main methods for detecting specific tumor-infiltrating immune cells included immunohistochemistry (IHC), tissue microarray (TMA) and immunofluorescence (IF). The most frequently used cut-off values to distinguish positive and negative (high and low) tumor infiltration was the median level, mean level or a certain specific value determined by counting under the microscope.

### Tumor-infiltrating lymphocytes

#### Subset of CD3+ T lymphocytes

**Figure 2:**
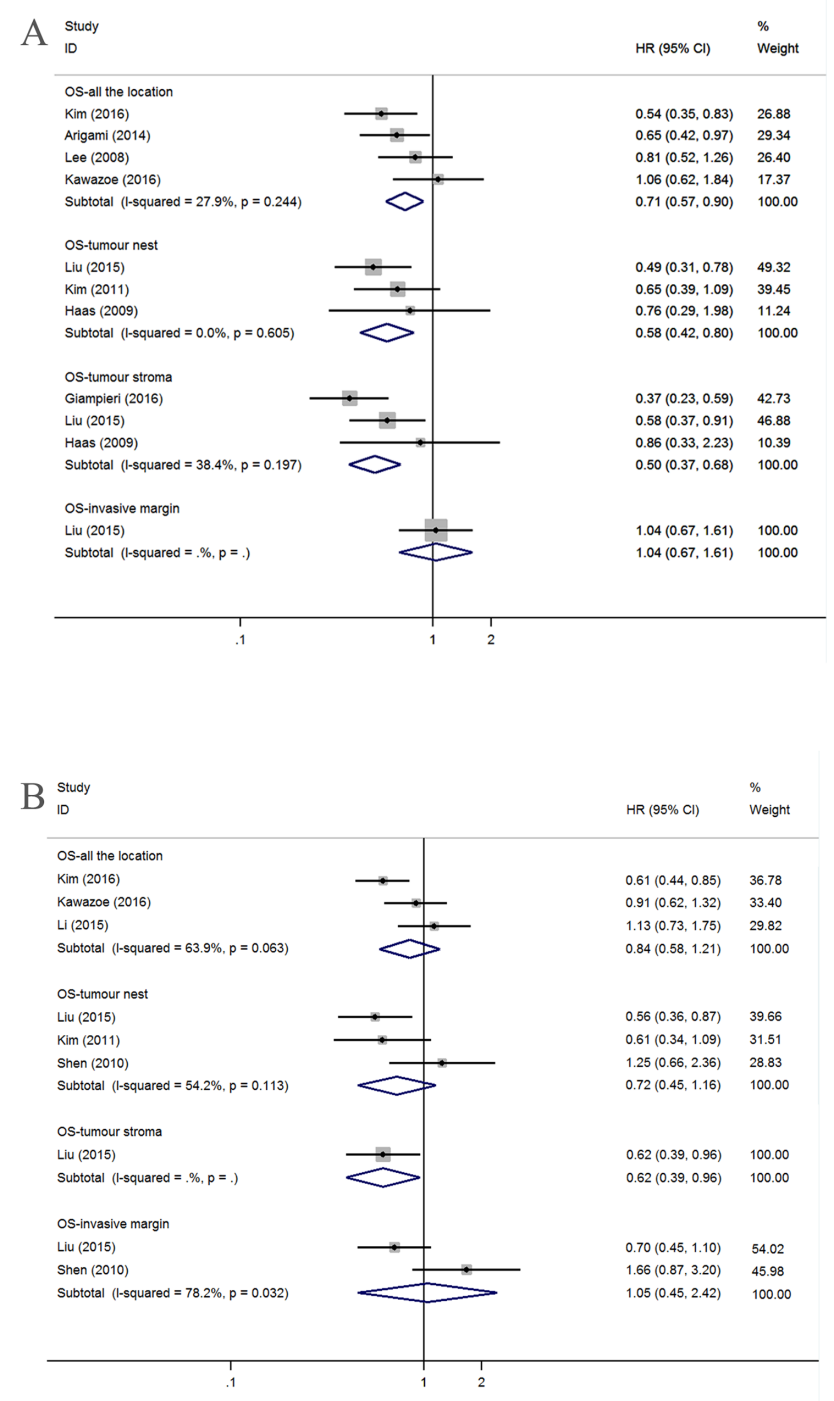
Forest plots of HRs to assess the correlation between prognosis and tumor-infiltrating immune cells **(A)** OS and CD3^+^, **(B)** OS and CD4^+^.

Eight articles that focused on the correlation between the infiltration of CD3+ T lymphocytes and the overall survival of GC patients were divided into eleven studies according to the location of tumor infiltration. Among these eleven studies, three, three, one, and four studies reported the infiltration of CD3+ T lymphocytes into the TN, the TS, the TM and AG, respectively. The estimated pooled HRs of OS for AG, TN, TS, and TM were 0.71 (95% confidence interval (CI)=0.57-0.90; *I*^*2*^=27.9%, *P*=0.244), 0.58 (95% CI=0.42-0.80; *I*^*2*^=0.0%, *P*=0.605), 0.50 (95% CI=0.37-0.68; *I*^*2*^=38.4%, *P*=0.197), and 1.04 (95% CI=0.67-1.61), respectively (Figure 2A). The above results indicate that better OS was associated with CD3+ T lymphocyte infiltration in AG, TN, and TS. Only two articles provided the relationship between the DFS/RFS and CD3+ T lymphocytes. DFS/RFS HRs of the two studies were as follows: AG: HR=0.62, 95% CI=0.40-0.98 and TN: HR=0.70, 95% CI=0.43-1.15 (data not shown).

#### Subset of CD4+ T lymphocytes

Six articles detected CD4+ T lymphocytes and investigated their relationship with prognostic value. Similarly, we grouped the six articles into nine studies involving OS and two studies involving DFS/RFS according to the location of infiltration. Because the heterogeneity was obvious, we used the random-effects model to estimate the HRs. OS was not associated with infiltration into a particular location, such as AG (n=3; HR=0.84, 95% CI=0.58-1.21; *I*^*2*^=63.9%, *P*=0.063), the TN (n=3; HR=0.72, 95% CI=0.45-1.16; *I*^*2*^=54.2%, *P*=0.113) or the TM (n=2; HR=1.05, 95% CI=0.45-2.42; *I*^*2*^=78.2%, *P*=0.032) (Figure 2B). Among the remaining three studies, one study assessed the relationship between OS and CD4+ T lymphocyte infiltration in TS (HR=0.62, 95% CI=0.39-0.96), and two studies involving DFS/RFS investigated the AG (HR=0.58, 95% CI=0.40-0.84) and TN (HR=0.71, 95% CI=0.41-1.24) (data not shown).

#### Subset of CD8+ T lymphocytes

**Figure 3:**
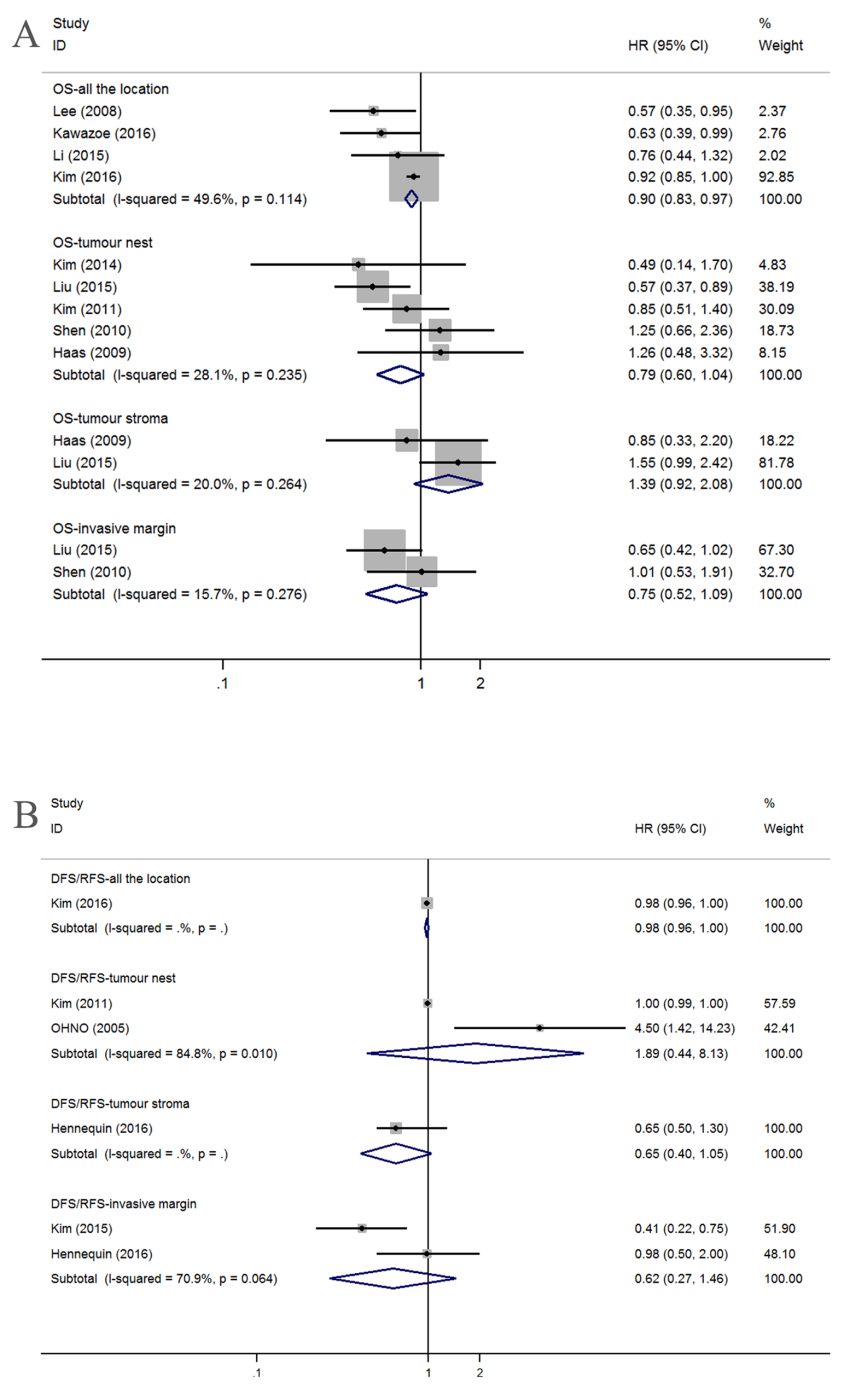
Forest plots of HRs to assess the correlation between prognosis and tumor-infiltrating immune cells **(A)** OS and CD8+, **(B)** DFS/RFS and CD8+.

By applying the aforementioned methods, we obtained 13 studies that investigated OS; after dividing these studies according to location, there were four, five, two, and two studies that addressed AG, the TN, the TS and the TM, respectively. We found that a high density of tumor-infiltrating CD8+ lymphocytes counted in AG was associated with good OS (HR=0.90, 95% CI=0.83-0.97, *I*^*2*^=49.6%, *P*=0.114) but that OS was not correlated with specific infiltration locations, such as the TN (HR=0.79, 95% CI=0.60-1.04; *I*^*2*^=28.1%,*P*=0.235), the TS (HR=1.39, 95% CI=0.92-2.08; *I*^*2*^=20.0%, *P*=0.264) or the TM (HR=0.75, 95% CI=0.52-1.09; *I*^*2*^=15.7%, *P*=0.276) (Figure 3A).

Six studies provided HRs and 95% CIs for the correlation between CD8+ T lymphocytes and DFS/RFS, with one study considering the AG (HR=0.98, 95% CI=0.96-1.00), two considering the TN (HR=1.89, 95% CI=0.44-8.13; *I*^*2*^=84.8%, *P*=0.010), one considering the TS (HR=0.65, 95% CI=0.40-1.05) and two considering the TM (HR=0.62, 95% CI=0.27-1.46; *I*^*2*^=70.9%, *P*=0.064) (Figure 3B).

#### Subset of Foxp3+ Treg lymphocytes

**Figure 4:**
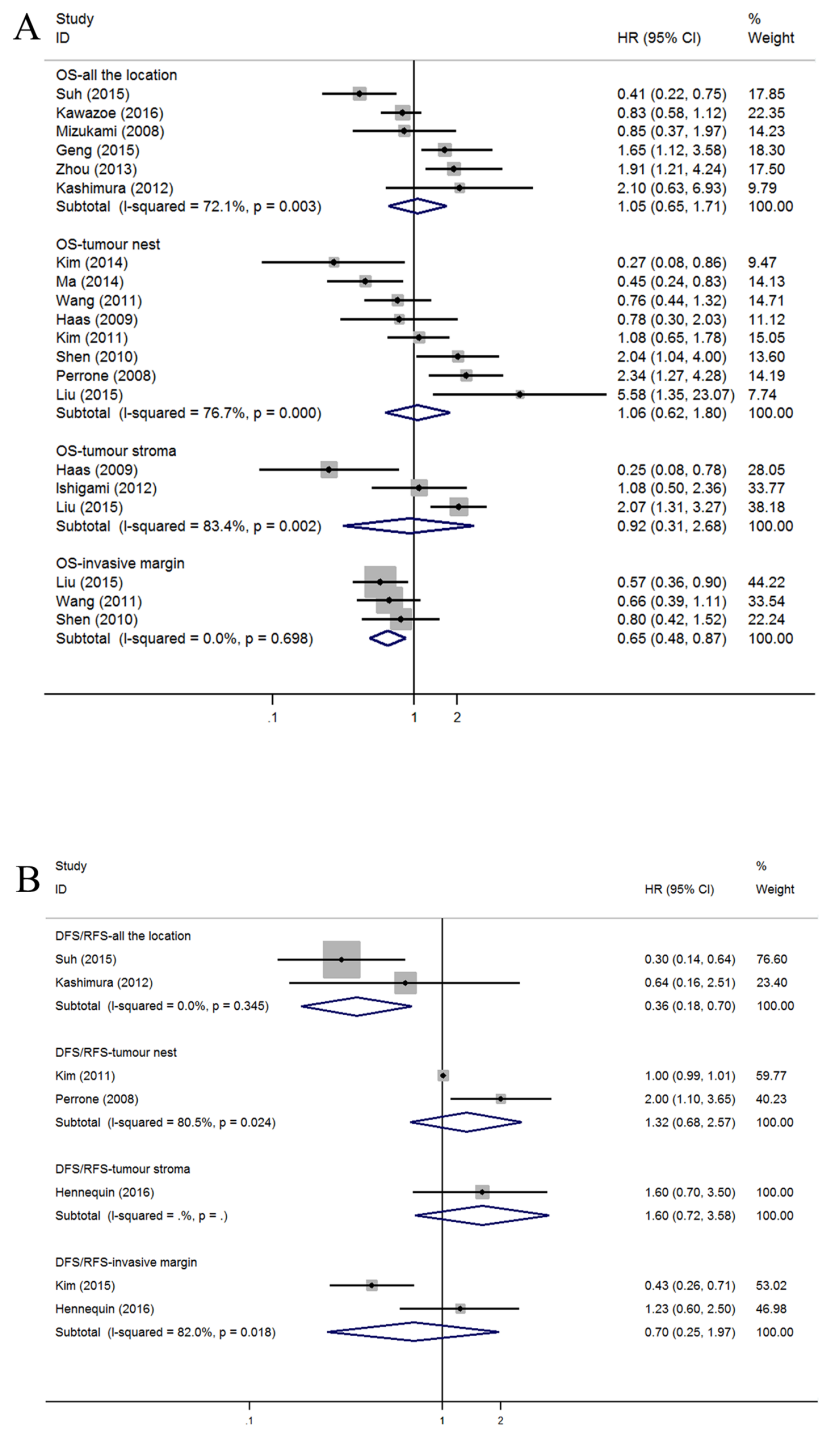
Forest plots of HRs to assess the correlation between prognosis and tumor-infiltrating immune cells **(A)** OS and FoxP3+, **(B)** DFS/RFS and FoxP3+.

Twenty studies concerning OS were obtained by splitting sixteen articles with regard to Foxp3+ Treg lymphocytes. No relationships were found between OS and AG (n=6; HR=1.05, 95% CI=0.65-1.71), TN (n=8; HR=1.06, 95% CI=0.62-1.80), or TS (n=3; HR=0.92, 95% CI=0.31-2.68). Significant heterogeneity was observed for AG (*I*^*2*^=72.1%, *P*=0.003), TN (*I*^*2*^=76.7%, *P*<0.001), and TS (*I*^*2*^=83.4%, *P*=0.002). However, GC patients with high tumor margin infiltration have better OS (n=3; HR=0.65, 95% CI=0.48-0.87) and no heterogeneity (*I*^*2*^=0.0%, *P*=0.698) (Figure 4A).

The high density of foxp3+ Treg cells in the AG indicated a better DFS/RFS (n=2; HR=0.36, 95% CI=0.18-0.70; *I*^*2*^=0.0%, *P*=0.345), and no association was found with limited studies between DFS/RFS and other tumor infiltration locations, including TN (n=2; HR=1.32, 95% CI=0.68-2.57; *I*^*2*^=80.5%, *P*=0.024), TS (n=1; HR=1.60, 95% CI=0.72-3.58), and TM (n=2; HR=0.70, 95% CI=0.25-1.97; *I*^*2*^=82.0%, *P*=0.018) (Figure 4B).

### Tumor-associated macrophages

#### CD68+ TAM

**Figure 5:**
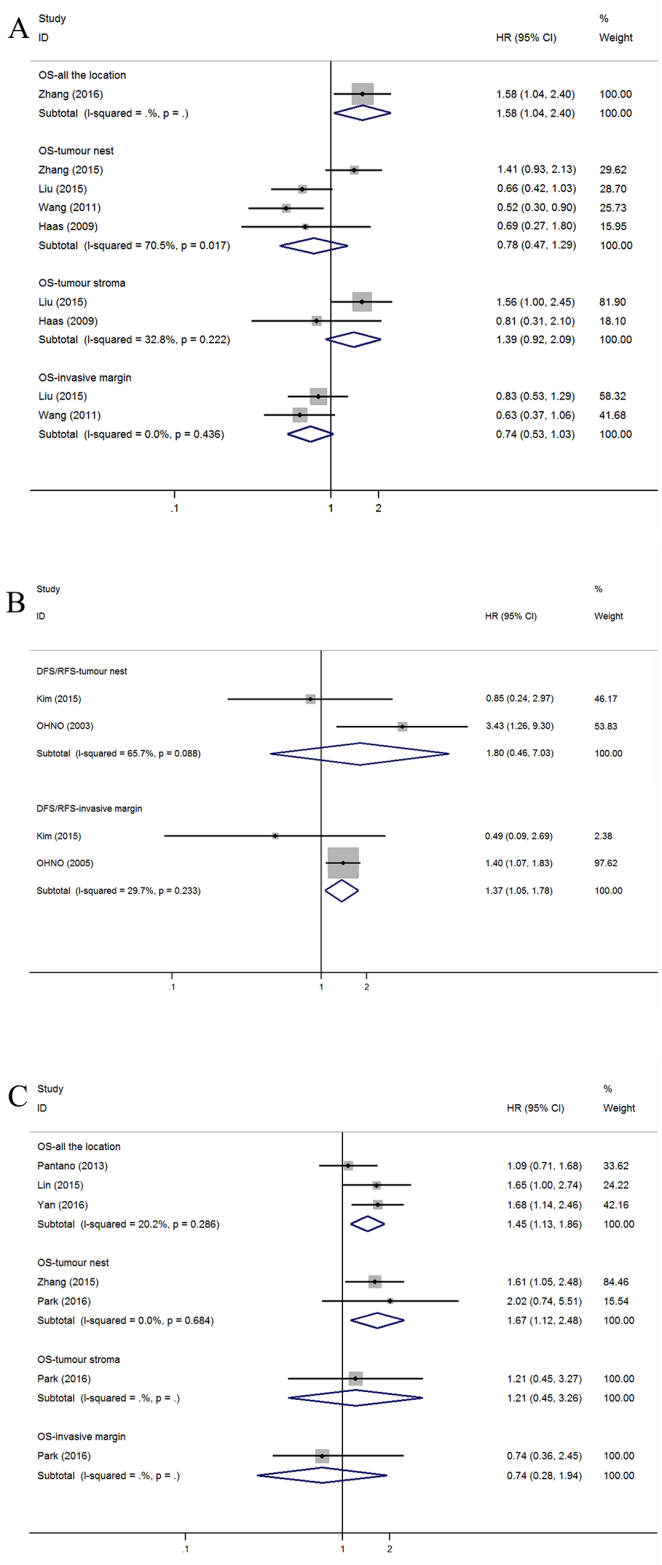
Forest plots of HRs to assess the correlation between prognosis and tumor-infiltrating immune cells **(A)** OS and M, **(B)** DFS/RFS and M, **(C)** OS and M2.

One study investigating the AG showed that the OS HR was 1.58 (95% CI=1.04-2.40). No correlations were found between OS and TN (n=4; HR=0.78, 95% CI=0.47-1.29; *I*^*2*^=70.5%, *P=*0.017), TS (n=2; HR=1.39, 95% CI=0.92-2.09; *I*^*2*^=32.8%, *P=*0.222) or TM (n=2; HR=0.74, 95% CI=0.53-1.03; *I*^*2*^=0.0%, *P*=0.436) (Figure 5A).

For the five studies that assessed DFS/RFS, the pooled HRs for different infiltrating locations in TN and TM were 1.80 (n=2, 95% CI=0.46–7.03) and 1.37 (n=2, 95% CI=1.05–1.78), respectively (Figure 5B).

#### Subset of M2 TAM.

Due to insufficient studies, we do not present the detailed pooled result of the M1. However, we drew the conclusion that worse OS is correlated with high M2 macrophage infiltration in AG (n=3; HR=1.45, 95% CI=1.13-1.86; *I*^*2*^=20.2%, *P*=0.286) and the TN (n=2; HR=1.67, 95% CI=1.12-2.48; *I*^*2*^=0.0%, *P*=0.684) but not the TM (n=1; HR=0.74, 95% CI=0.28-1.94) or the TS (n=1; HR=1.21, 95% CI=0.45-3.26) (Figure 5C).

#### Tumor-associated macrophages and clinicopathological characteristics

When sufficient data were available from original articles, correlations between TAM infiltration and patients’ clinicopathological characteristics were evaluated by pooling extracted data (Table 3). There was no relationship between CD68+ TAMs in the TN and gender (female vs male), tumor size (<4 m vs >4 cm), T stage (T_1_+T_2_ vs T_3_+T_4_), N stage (N_0_ vs N_1-3_) or TNM stage (I+II vs III+IV). However, male (n=2; OR=2.05, 95% CI=1.31-3.21; *I*^*2*^=0.0%, *P=*0.663) and N_1-3_ (n=2, OR=2.57, 95% CI=1.11-5.93; *I*^*2*^=67.5%, *P=*0.080) patients have high densities of M2 TAMs in AG, although tumor size (<5 cm vs >5 cm) was not associated with the density of M2 TAMs in AG. However, in the TN, male patients (n=2, OR=0.55, 95% CI=0.32-0.92; *I*^*2*^=0.0%, *P=*0.781) had a low density of M2 TAMs. No associations were found between T stage (T_1_+T_2_ vs T_3_+T_4_), N Stage (N_0_ vs N_1-3_) and TNM Stage (I+II vs III+IV).

**Table 2:** The pooled relationships between tumor-infiltrating immune cells subsets and the prognosis of patients with gastric cancer.

Subset/Outcome	Location	No. Of Studies	HR(95%CI)	Model	Heterogeneity		Publication bias	
					I^2^	P value	Begg’s P	Egger’s P
CD3								
OS	AG	4	0.71(0.57,0.90)	Fixed	27.9%	0.244	0.308	0.221
	TN	3	0.58(0.42,0.80)	Fixed	0.00%	0.605	1	0.49
	TS	3	0.50(0.37,0.68)	Fixed	38.4%	0.197	1	0.589
	TM	1	1.04(0.67,1.61)	-	-	-	-	-
CD4								
OS	AG	3	0.84(0.58,1.21)	Random	63.9%	0.063	0.296	0.125
	TN	3	0.72(0.45,1.16)	Random	54.2%	0.113	0.296	0.424
	TS	1	0.62(0.39,0.96)	-	-	-	-	-
	TM	2	1.05(0.45,2.42)	Random	78.2%	0.032	-	-
CD8								
OS	AG	4	0.90(0.83,0.97)	Random	49.6%	0.114	0.734	0.07
	TN	5	0.79(0.60,1.04)	Fixed	28.1%	0.235	0.806	0.661
	TS	2	1.39(0.92,2.08)	Fixed	20.0%	0.264	-	-
	TM	2	0.75(0.52,1.09)	Fixed	15.7%	0.276	-	-
DFS/RFS	AG	1	0.98(0.96,1.00)	-	-	-	-	-
	TN	2	1.89(0.44,8.13)	Random	84.8%	0.010	-	-
	TS	1	0.65(0.40,1.05)	-	-	-	-	-
	TM	2	0.62(0.27,1.46)	Random	70.9%	0.064	-	-
FoxP3								
OS	AG	6	1.05(0.65,1.71)	Random	72.1%	0.003	0.707	0.526
	TN	8	1.06(0.62,1.80)	Random	76.7%	<0.001	1	0.889
	TS	3	0.92(0.31,2.68)	Random	83.4%	0.002	-	-
	TM	3	0.65(0.48,0.87)	Fixed	0.0%	0.698	0.296	0.038
DFS/RFS	AG	2	0.36(0.18,0.70)	Fixed	0.0%	0.345	-	-
	TN	2	1.32(0.68,2.57)	Random	80.5%	0.024	-	-
	TS	1	1.60(0.72,3.58)	-	-	-	-	-
	TM	2	0.70(0.25,1.97)	Random	82.00%	0.018	-	-
M								
OS	AG	1	1.58(1.04,2.40)	-	-	-	-	-
	TN	4	0.78(0.47,1.29)	Random	70.5%	0.017	0.734	0.581
	TS	2	1.39(0.92,2.09)	Fixed	32.8%	0.222	-	-
	TM	2	0.74(0.53,1.03)	Fixed	0.0%	0.436	-	-
DFS/RFS	TN	2	1.80(0.46,7.03)	Random	65.7%	0.088	-	-
	TM	2	1.37(1.05,1.78)	Fixed	29.7%	0.223	-	-
M2								
OS	AG	3	1.45(1.13,1.86)	Fixed	20.2%	0.286	1	0.972
	TN	2	1.67(1.12,2.48)	Fixed	0.0%	0.684	-	-
	TS	1	1,21(0.45,3.26)	-	-	-	-	-
	TM	1	0.74(0.28,1.94)	-	-	-	-	-
CD45RO								
OS	AG	2	0.56(0.37,0.84)	Fixed	0.0%	0.526	-	-
CD57								
OS	TN	2	0.59(0.44,0.79)	Fixed	0.0%	0.420	-	-
Granzyme B								
OS	TN	2	0.81(0.51,1.29)	Fixed	0.0%	0.838	-	-
Dendritic cell								
OS	AG	3	0.62(0.15,2.53)	Random	84.4%	0.002	-	-

Abbreviations: AG=all locations, TN=tumor nest, TS=tumor stroma, TM=tumor invasive margin, OS=overall survival, DFS=disease-free survival, RFS=relapse-free survival.

**Table 3:** Correlations between tumor associated macrophages (TAMs) and clinicopathological characteristics.

Clinicopathological characteristics	No of studies	OR	Confident interval	Model	heterogeneity	
					*I*^*2*^	*P*
Tumor nest CD68+ TAMs and clinicopathological characteristics						
Gender (female VS male)	3	0.87	0.41-1.82	Random	69.2%	0.039
Tumor size (<4cm VS >4cm)	2	0.91	0.57-1.45	Fixed	0.0%	0.433
T stage (T_1_+T_2_ VS T_3_+T_4_)	2	1.20	0.74-1.96	Fixed	0.0%	0.346
N Stage(N_0_ VS N_1-3_)	3	1.32	0.45-3.91	Random	82.6%	0.003
TNM Stage (I+II VS III+IV)	2	1.04	0.34-3.91	Random	84.8%	0.010
All the locations M2 TAMs and clinicopathological characteristics						
Gender (female VS male)	2	2.05	1.31-3.21	Fixed	0.0%	0.663
Tumor size (<5cm VS >5cm)	2	1.11	0.71-1.73	Fixed	0.0%	0.647
N stage (N0 VS N_1-3_)	2	2.57	1.11-5.93	Random	67.5%	0.080
Tumor nest M2 TAMs and clinicopathological characteristics						
Gender (female VS male)	2	0.55	0.32-0.92	Fixed	0.0%	0.781
T stage (T_1_+T_2_ VS T_3_+T_4_)	2	1.41	0.84-2.36	Fixed	0.0%	0.341
N Stage(N_0_ VS N_1-3_)	2	1.68	1.02-2.78	Fixed	0.0%	0.882
TNM Stage (I+II VS III+IV)	2	1.39	0.84-2.28	Fixed	0.0%	0.743

### Other cells

**Figure 6:**
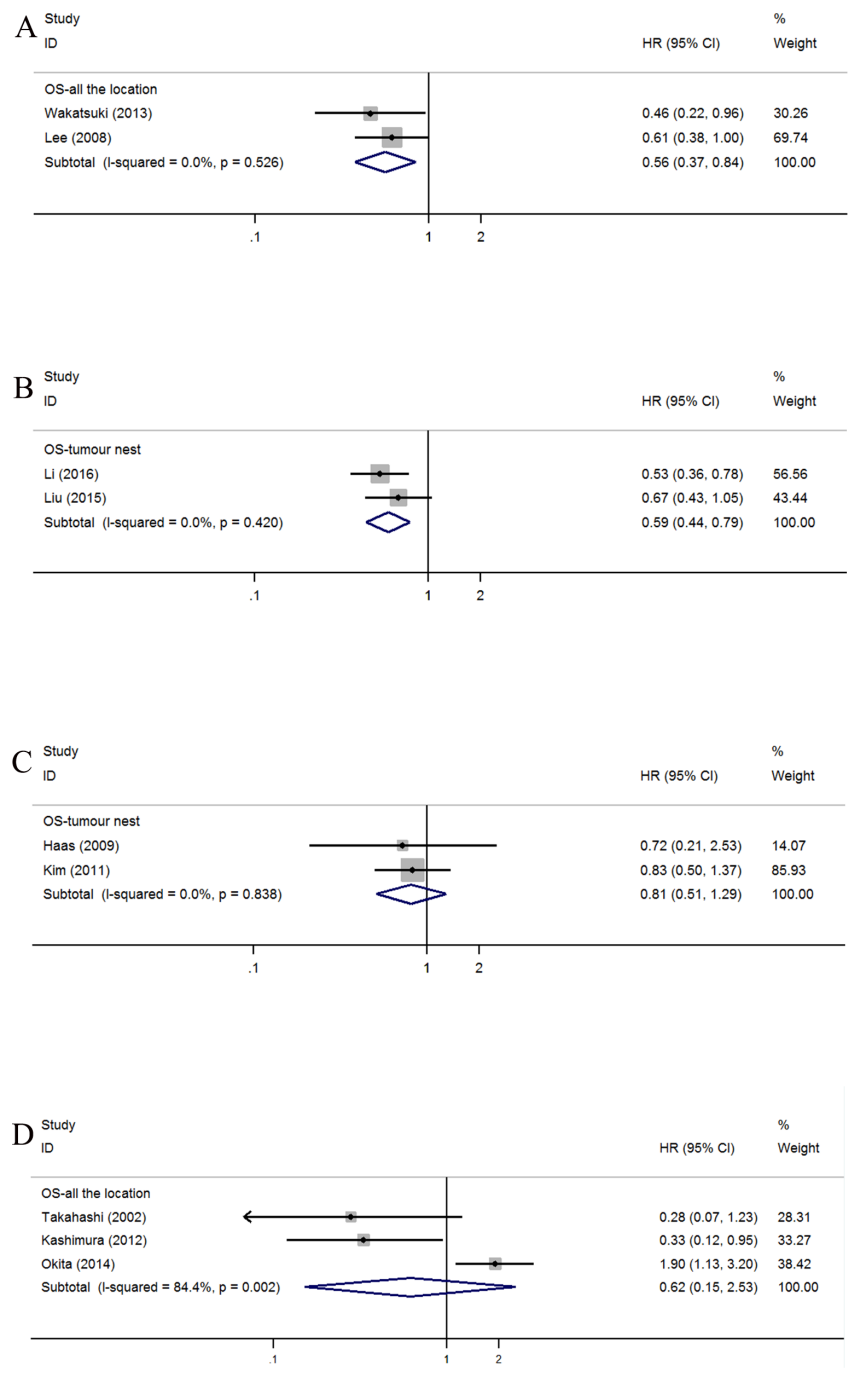
Forest plots of HRs to assess the correlation between prognosis and tumor-infiltrating immune cells **(A)** OS and CD45RO^+^, **(B)** OS and CD57^+^, **(C)** OS and Granzyme B **(D)** OS and Dendritic cell

Due to the limited number of studies, we optionally presented the pooled OS of certain cell subsets, such as CD45RO+ cells in AG (n=2; HR=0.56, 95% CI=0.37-0.84; *I*^*2*^=0.0%, *P*=0.526) (Figure 6A), CD57+ natural killer cells in TN (n=2; HR=0.59, 95% CI=0.44-0.79; *I*^*2*^=0.0%, *P=*0.420) (Figure 6B), granzyme B+ cells in TN (n=2; HR=0.81, 95% CI=0.51-1.29; *I*^*2*^=0.0%, *P*=0.838) (Figure 6C), and dendritic cells in AG (n=3; HR=0.62, 95% CI=0.15-2.53; *I*^*2*^=84.4%, *P*=0.002) (Figure 6D). Nevertheless, additional studies should be analyzed to determine the reproducibility of these results.

### Subgroup and sensitivity analysis

Because obvious heterogeneity was found in the TN group of FoxP3+ Treg cells, subgroup analyses were conducted to seek the source of this heterogeneity. Ethnicity, publication year, score, tumor stage and identification number were adopted as the basis for grouping (Table 4). In the group “publication before 2011,” worse OS was associated with high level of FoxP3+ Treg lymphocytes (HR=1.82, 95% CI=1.21-2.74; *I*^*2*^=47.10%, *P=*0.151). However, heterogeneity was still significant in other subgroups (Table 4). No individual study could alter the overall trend when it was removed from the meta-analysis of Foxp3+ cell infiltration in the TN panel.

**Table 4:** Subgroup analysis of correlation between prognosis and FoxP3+ Treg cell infiltration in the TN

Subgroup	No of study	HR(95%CI)	Heterogeneity	
			*I*^*2*^	*P*
Region				
Asia	6	0.95(0.52,1.76)	77.20%	0.001
Europe	2	1.44(0.49,4.20)	72.30%	0.057
Publication year				
After 2011	5	0.80(0.42,1.52)	73.80%	0.004
Before 2011	3	1.82(1.21,2.74)	47.10%	0.151
Score				
≥6	6	1.07(0.60,1.89)	68.8%	0.007
<6	2	1.03(0.20,5.17)	92.9%	<0.001
Stage				
I-III	2	1.42(0.76,2.65)	54.80%	0.137
I-IV	5	0.74(0.37,1.46)	68.90%	0.012
II-III	1	2.34(1.27,4.30)	-	-
Patients’ number				
≥120	4	0.85(0.38,1.93)	78.0%	0.003
<120	4	1.32(0.62,3.03)	81.7%	0.001

### Publication bias

**Figure 7:**
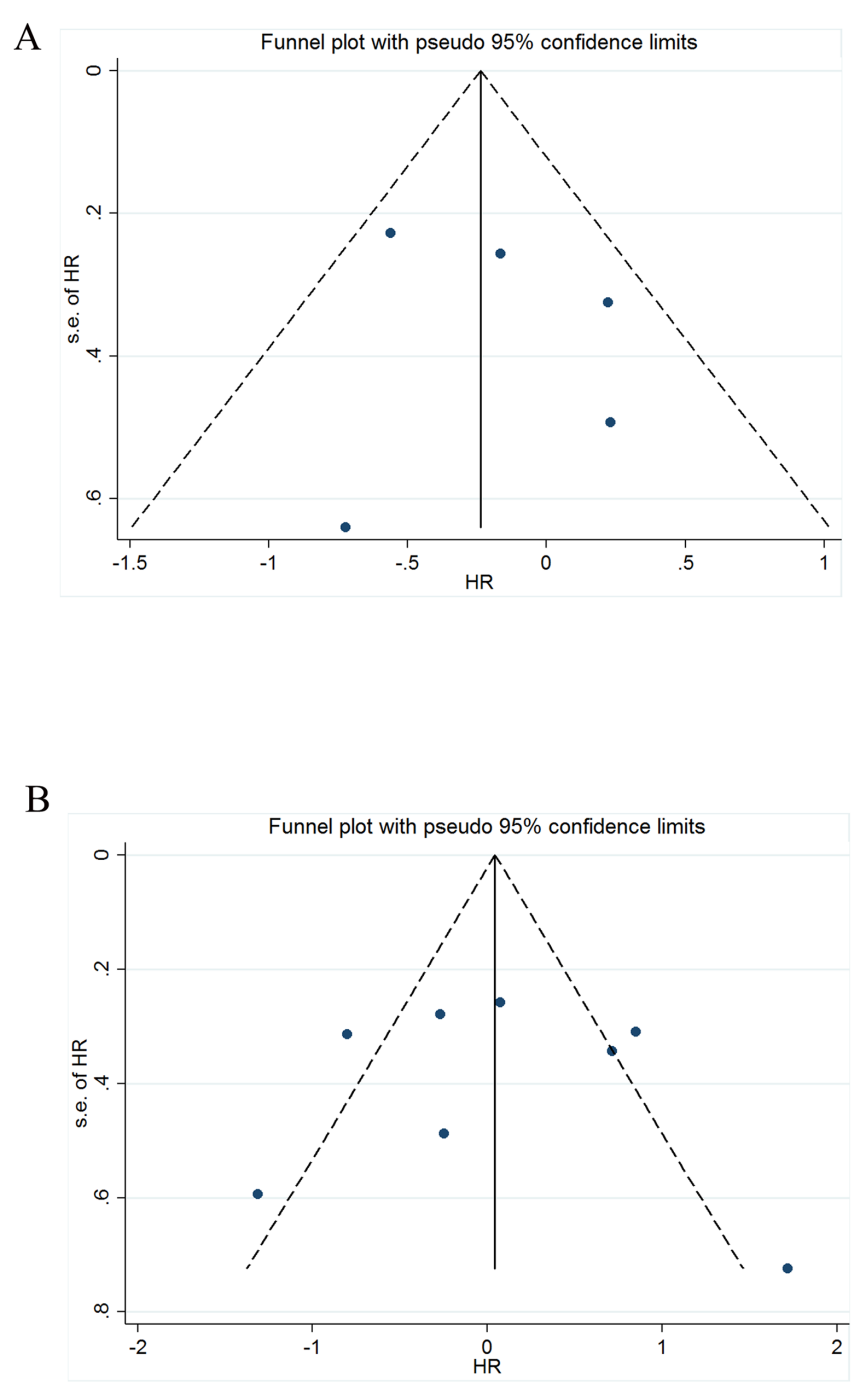
Funnel plot of the meta-analysis **(A)** OS and CD8+ infiltration in TN, **(B)** OS and FoxP3 infiltration in the TN

The funnel plots of the CD8+ T cell infiltration in TN (Figure 7A) and FoxP3+ Treg cells (Figure 7B) were substantially symmetric. The *P* values of Egger’s and Begg’s tests in the other panels were all greater than 0.05, except for FoxP3+ Treg cell infiltration in TM (Begg’s *P*=0.038) (Table 2).

## DISCUSSION

Tumor-infiltrating immune cells can influence the prognosis of cancer patients by directly or indirectly participating in immune responses and angiogenesis. For example, dendritic cells (DCs) can capture and present antigens released by tumor cells; effector T cells (CD8+) and TAMs can dissolve and devour tumor cells; and helper T cells (CD4+), including FoxP3 Tregs, impose restrictions on immune response [50]. There are two subgroups of TAMs: M1 cells and M2 cells. M1 TAMs promote inflammatory responses and antitumor activity, whereas M2 TAMs inhibit inflammatory responses and enhance tumor progression by promoting angiogenesis and epithelial-mesenchymal transition (EMT) [51].

This meta-analysis was performed to investigate the relationship between the clinical outcome and density of tumor-infiltrating immune cells in different locations such as TN, TS and TM. The results reveal that the high density of CD3+ T cell infiltration in AG, TN, and TS is associated with better OS. Similarly, high densities of CD8+ T cells in AG and FoxP3+ Tregs in the TM predict better OS, and a high density of FoxP3+ Tregs infiltrated into AG is associated with better DFS/RFS. Meanwhile, CD45RO+ cells in AG and CD57+ natural killer cells in TN are also associated with better OS. In contrast, TAMs (CD68+) in the TM may negatively affect DFS/RFS.

It is interesting that the prognosis of the same immune cells can vary according to different locations of infiltration. For example, a high density of CD8+ T cells in the AG is associated with better OS and has no predictive effect on prognosis in TN, TS and TM. The tumor microenvironment varies in different locations, which may cause differences of the functions of the same immune cell. The TN is mainly composed of tumor cells, which are antigens for immune cells. Tumor cells can exhaust T cells by expressing coinhibitory molecules, such as CTLA-4 and PD-L1 [52]. However, in the TS, microvessels and fibroblasts are the main support components for promoting angiogenesis and tumor metastasis, and the function of immune cells can be limited by TS components [3]. Therefore, it is not surprising that in a previous meta-analysis, a high density of foxp3+ Treg cells benefited from 1-, 3-, and 5-year OS after surgical resection [53]. However, when stratifying according to infiltrating locations, no predictive relationships were found between OS and FoxP3+ Treg cells in different infiltrating locations, such as the TN. Galon et al [16, 54] suggested that this can improve the accuracy of the prediction of patients’ survival by the combined analysis of tumor-infiltrating regions, and it is important to take the effect of tumor microenvironment into consideration.

However, summary HRs of certain locations show negative relationships between the density of immune cells and prognosis. This may result from the restriction of the number of available studies and the vast difference between the original results. For example, only three studies involved the infiltration of CD4+ T cells in TN, and one study suggested that the high density of CD4+ T cells can benefit OS [9]. However, two studies showed that CD4+ T cells are not associated with OS [32, 43]. Therefore, further studies that utilize uniform pathology standards are needed to support this conclusion.

The pooled results need to be examined from different perspectives because of several limitations. First, statistical errors are inevitable because some HRs of OS and DFS/RFS were obtained from Kaplan–Meier (KM) curves, though two researchers examined data from one curve to minimize the error. Second, vast differences resulting from different regions, genders, pathologic types, and status of microsatellite instability (MSI) may also influence the differences from the original results [22, 38, 41]. Third, we failed to include some potential studies that could have been extrapolated from other studies or conference abstracts without sufficient data.

In conclusion, the density of immune cells in different locations combined with histopathological evaluation can be used as a prognostic marker. With further research, the relationship between density, the location of tumor-infiltrating immune cells and GC patients’ clinical outcome will become clearer.

## MATERIALS AND METHODS

### Search strategy

We performed our meta-analysis by searching PubMed and Embase with a cut-off of September 2016. The search terms were as follows: (lymphocytes or immune cells) AND (gastric OR stomach) AND (survival OR prognosis OR prognostic). Abstracts and titles were read by two researchers who used the samecriteria to exclude irrelevant articles. The full texts of remaining articles were carefully screened to find all eligible articles to avoid unnecessary basis. Nonconformity between the two reviewers was resolved through discussions among all authors in this meta-analysis.

### Inclusion and exclusion criteria

We selected eligible articles in this meta-analysis according to the following criteria: (1) evaluation of the infiltration of immune cells, such as CD3+ lymphocytes, CD4+ lymphocytes, CD8+ lymphocytes, Foxp3+ Tregs, natural killer cells and macrophages, into primary gastric tumors; (2) examination of ≥50 samples; (3) evaluation by immunohistochemical staining (tissue microarrays) or immunofluorescence; and (4) presentation of OS or DFS or RFS values for high (positive) and low (negative) immune cell infiltration density that were either specifically stated or depicted using Kaplan–Meier curves.

We excluded the following articles: case reports, review articles, meta-analyses, animal studies, studies with duplicate cases, Epstein–Barr virus (EBV)-associated gastric cancer (EBVaGC), and studies or conference abstracts without sufficient data for the calculation of HR and 95% CI.

### Data extraction and study quality assessment

Two investigators independently extracted data from eligible studies. Data including author, journal, year of publication, sample size, stage of tumor, follow-up duration, immune cell subset, site of immune cells, cut-off point, outcome, hazard ratios, and 95% CIs were summarized. We evaluated the quality of each study using the criteria presented by De Graeff [55], which were derived from McShane et al [56] and Hayes et al [57]; details are shown in Supplementary Table 2.

### Statistical analysis

Integrated calculation of the extracted data in this meta-analysis was performed using Stata 14.0 software. For time-to-event outcomes, HRs along with 95% CIs were pooled to measure the correlation between tumor-infiltrating immune cell density and prognosis. When Kaplan–Meier curves were provided instead of HR, two researchers independently estimated the HR indirectly from the curves using Engauge Digitizer version 9.0 according to the methods described by Tierney et al [58, 59]. The chi-square test and *I*^*2*^ statistic were used to assess heterogeneity [60]. Heterogeneity was thought to exist when *P<*0.05 and/or *I*^2^>50%; in such cases, a random-effects model was used. Then, to identify the source of heterogeneity, subgroup analysis was employed. Publication bias was examined by performing Begg’s and Egger’s tests and evaluating the symmetry of the funnel plot [61].

### SUPPLEMENTARY MATERIALS TABLES




