# Supplementary material for: Tumor-infiltrating immune cells and prognosis in gastric cancer: a systematic review and meta-analysis
Source: Oncotarget. 2017 May 3;8(37):62312–29. doi: 10.18632/oncotarget.17602 (PMC5617507; doi:10.18632/oncotarget.17602)
Supplement: Supplementary file 1 [file oncotarget-08-62312-s001.pdf]

Tumor-infiltrating immune cells and prognosis in gastric cancer: a systematic review and meta-analysis

SUPPLEMENTARY MATERIALS

Supplementary Table 1: Baseline Characteristics of Included Studies of all studies included in the meta-analysis.

See Supplementary File 1

Supplementary Table 2: Criteria for quality assessment by De Graeff [de Graeff et al., 2009]

Criterium		Points
1. Is the population under study defined with in- and exclusion criteria?		**1**
2. Were patient data prospectively collected?		**1**
3. Are the main prognostic patient and tumour characteristics presented?[1]		**1**
4. Is the method used for determination of protein expression specified?		**2**
Criteria for immuhistochemistry / FISH:<br>• Is the immunohistochemical staining protocol specified?[2]<br>• Were stainings evaluated by > 1 observer?	1<br>1	
Criteria for mutational analysis:<br>• Is the PCR protocol specified?[3]<br>• Is the SSCP and/or sequencing protocol specified?	1<br>1	
Criteria for Southern Blot:<br>• Are the restriction enzymes used specified?<br>• Is the hybridization methods specified?[4]	1<br>1	
Criteria for EGF binding assay:<br>• Are positive and negative controls specified?<br>• Is the assay protocol specified?[5]	1<br>1	
Criteria for RT-PCR:<br>• Is the RNA isolation method and cDNA synthesis specified?<br>• Is the PCR protocol specified?[3]	1<br>1	
Criteria for enzyme immunoassay<br>• Is the antibody used specified?<br>• Are control samples and a cut-off value specified?	1<br>1	
5. Is the study endpoint defined?		**1**
6. Is the time of follow up specified?		**1**
7. Is loss during analysis or follow up described?		**1**
		**Max. 8 points**

[1]At least four of the following characteristics: age at diagnosis, FIGO stage, tumor type, differentiation grade and residual tumor after primary surgery. [2]At least four of the following criteria: antigen retrieval, primary antibody, dilution, detection method, cut-off value for positive expression: [3]At least the primers used and the annealing temperature or number of cycles [4]At least internal controls and probes used [5]At least four of the following criteria: label, incubation time, filter size, separation method (BSA / Tris-sucrose), cut-off value for positive expression
